# Measurement of Aortic Atherosclerotic Disease Severity: A Novel Tool for Simplified, Objective Disease Scoring Using CT Angiography

**DOI:** 10.7759/cureus.15561

**Published:** 2021-06-10

**Authors:** Priyanka Reddy, Madhurima R Chetan, Charles R Tapping, Luke Lintin

**Affiliations:** 1 Department of Radiology, Oxford University Hospitals National Health Service Foundation Trust, Oxford, GBR; 2 Department of Interventional Radiology, Oxford University Hospitals National Health Service Foundation Trust, Oxford, GBR; 3 Department of Radiology, Buckinghamshire Healthcare National Health Service Trust, Aylesbury, GBR

**Keywords:** atherosclerosis, vascular calcification, computed tomography (ct ), computed tomographic angiography, calcium scoring, abdominal aorta

## Abstract

Introduction

Vascular calcification is a recognized indicator of cardiovascular morbidity and mortality. Calcium scoring is a widely used tool to measure coronary artery calcification, however has limitations for use elsewhere in the body. There is currently no gold standard for quantifying abdominal aortic calcification (AAC).

We propose a simple and reproducible method to assess the severity of AAC using multiplanar reconstruction (MPR) in CT angiograms (CTA).

Methods

A retrospective analysis of CTAs from 75 patients over two years was performed. Using a novel six-point scoring system, three radiologists independently scored the severity of AAC in the distal abdominal aorta. Interclass correlation (ICC) was used to assess the degree of agreement between the three raters.

Calcium scoring of the same region was also calculated for each patient. We used Spearman's rank correlation coefficient to compare the CT calcium score with the corresponding average rater's atheroma score.

Results

There was significant agreement between raters’ scores, with an ICC value = 0.972, 95% (CI 0.959-0.981, p < 0.0001). There was also a strong correlation between an average rater's atheroma score with the corresponding CT calcium score, rho = 0.85 (p < 0.0001).

Conclusion

The results show excellent reproducibility of scores between radiologists, as well as a strong correlation between this novel scoring tool and calcium scores, indicating that it is a reliable method for the grading of AAC.

We propose that this simple semi-quantitative method could form a widely used system for AAC disease stratification.

## Introduction

This article has been previously presented as an electronic poster at the CIRSE 2020 summit on September 12-16, 2020.

Vascular calcification (a sign of atherosclerotic disease) is a frequently encountered imaging finding on plain film and cross-sectional studies and is a recognized marker of cardiovascular morbidity and mortality [1,2]. It has a multifactorial etiology and the presence, extent, and severity of the disease are known to have a positive correlation with increased cardiovascular events [3]. This risk is known to be higher in patients with chronic renal disease [3,4,5]. 

A common practice for radiologists is to describe the severity of abdominal aortic disease based on subjective interpretation (e.g., “mild, moderate, severe”). There is a lack of standardization which makes this assessment prone to significant inter-observer variability. To date, there is no proposed tool for quantifying aortic calcification using CT.

A study by Kauppila et al. was one of the first to describe a system to quantify abdominal aortic calcification (AAC) in a subgroup of 617 patients from the Framingham Heart Study [6]. The authors measured visible calcific disease on lateral lumbar radiographs to develop a grading system and calculate an AAC score. Although not used in clinical practice, this is the only validated tool that has been reproduced in later studies such as that by Honkanen et al. and Grant et al. [4,7]. Authors in later studies largely focused on a select population of patients with end-stage renal disease and who are naturally at risk of atherosclerotic disease.

Currently, quantifying calcific vascular disease is a common use of cardiac CT, whereby calcium scoring is the gold standard in measuring coronary artery calcification [3]. This plays a vital role in the assessment pathway of cardiovascular disease risk stratification, whereby many scoring systems are available which utilize the generated score [8]. One disadvantage of calcium scoring software is that it can be time-consuming and not easily applicable to other vessels in the body.

The aforementioned studies that have focused on the abdominal aorta, have suggested scoring utilizing plain radiographs [5,6,7]. It can be argued that this is a dose-saving modality compared to CT. However, the exact extent of vessel calcification is not always visible on plain films and is dependent on the quality of the radiograph - which is, in turn, dependent on many factors (patient body habitus, variation in exposure rates, overlying obscuring bowel gas, etc). This can therefore impede accurate assessment of disease. Visualization of vessel calcification on CT is far superior than on plain radiographs.

CT is now readily available and a widely used modality in the imaging world for a range of indications. To date, there is no universally acknowledged system for measuring AAC. This paper proposes a novel method for semi-quantitative AAC scoring using CT angiography (CTA) with the intention to provide a simple and reproducible assessment of vascular calcific disease, negating the need for a dedicated workstation with calcium scoring functionality.

## Materials and methods

A retrospective analysis was conducted over a two-year timeframe from May 2016 to June 2018. The study population consisted of 75 male patients aged between 54 and 94 years from a single center who had already undergone CTA for pre-procedural planning prior to prostate artery embolization.

For the purpose of reproducibility, the segment of the abdominal aorta extending from the inferior mesenteric artery (IMA) to the level of bifurcation into the common iliac arteries was analyzed.

Images were reviewed using multiplanar reconstruction software (InSight PACS, Insignia Medical Systems, UK). Sagittal reformats centered on the middle of the lower abdominal aorta were viewed on “bone windows” - window width: 2500 HU (Hounsfield units), window length: 200 HU - and a maximum intensity projection (MIP) of 20 mm was applied to clearly demonstrate the degree of calcification in the anterior and posterior aortic walls. The fraction of atheromatous (calcified) wall to disease-free wall was then documented for both the anterior and posterior aortic walls.

**Figure 1:**
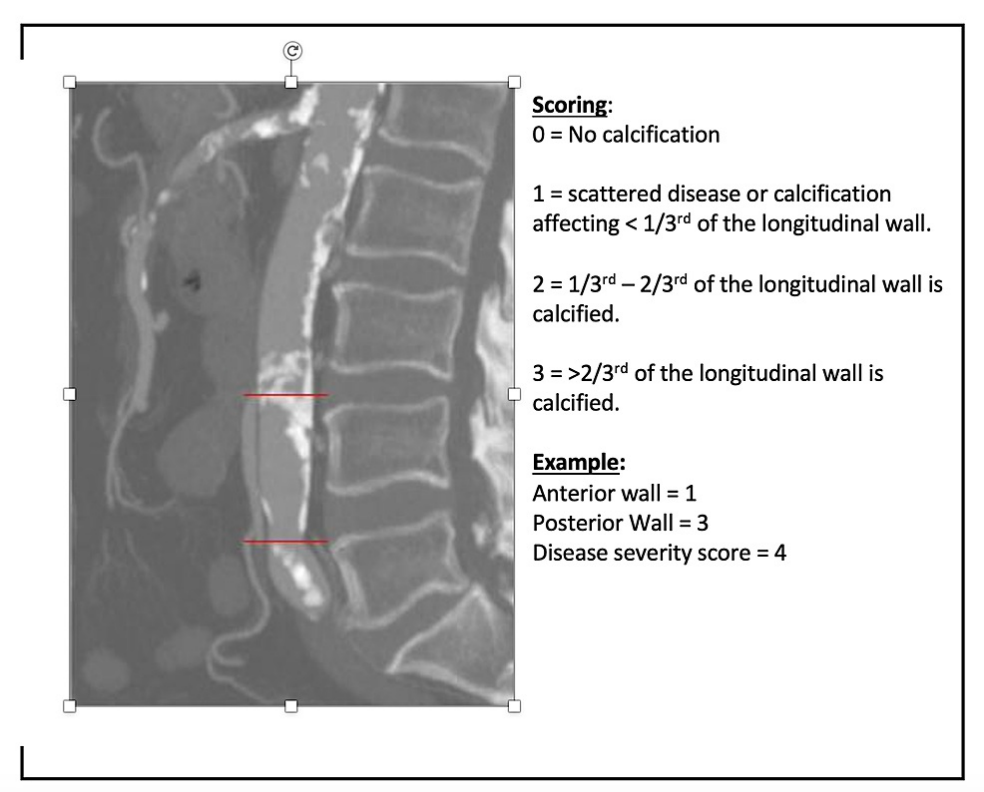
An applied example of disease grading in the determined segment with a combined score from the anterior and posterior walls.

Disease severity in the selected sagittal segment was calculated from a combination of two scores: 1) Anterior wall score of 0-3, and 2) Posterior wall score of 0-3. A maximum score of 6 indicates severe disease. A score of 0 was given if there was no calcification present. A score of 1 was given if less than a third of the longitudinal length of the wall was calcified. A score of 2 was given if between one-third and two-thirds of the longitudinal length was calcified and a score of 3 was given if more than two-thirds of the length of the wall was calcified. The scores from the anterior and posterior walls are then summed to give the disease severity score (ranging between 0 and 6). Figure 1 demonstrates a schematic representation of the scoring system and an applied example. The image depicts a reformatted sagittal slice from a CTA, highlighting the abdominal aortic segment between the IMA and common iliac bifurcation. 

Three radiologists of different grades; consultant, senior radiologist, and junior radiologist with 10, five, and two years of experience (respectively), separately scored the same 75 scans using the system described earlier. All radiologists were blinded to each others’ scores. 

**Figure 2:**
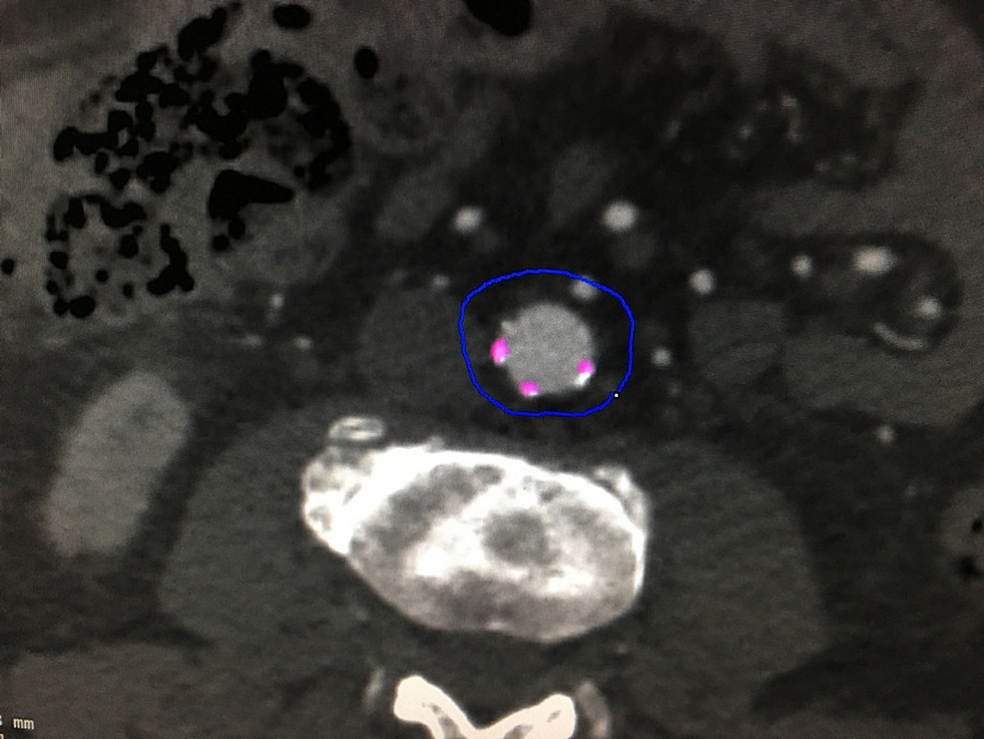
Calcium scoring using Aquarius workstation; TeraRecon software. Axial CT slice demonstrating selection of the plaques (which are highlighted in pink).

A calcium score was also calculated once for each patient at the same aortic segment, using a dedicated vascular workstation (Aquarius; TeraRecon, San Mateo, CA, USA). A minimum threshold of 450 HU and a maximum of 3070 HU were used for the calcium detection settings in the software. The calcified plaques in the aorta were then individually selected between the IMA origin and the bifurcation into the iliac vessels to generate a total calcium score, see Figure 2. In subjects with confluent calcification (extensive disease), the software often included calcification outside the defined aortic segment due to the nature of calcium score calculation. In these circumstances, the score was simply accepted as it is not possible to manually limit the region being assessed in a craniocaudal direction.

Statistical Analysis

Statistical analysis was conducted using SPSS software.

Interclass Correlation (ICC) was used to assess the consistency of atheroma scores (rater score) between each radiologist for every patient. 

An average of the three rater scores for each patient was then correlated with the corresponding calcium score using Spearman's rank correlation coefficient.

## Results

**Figure 3:**
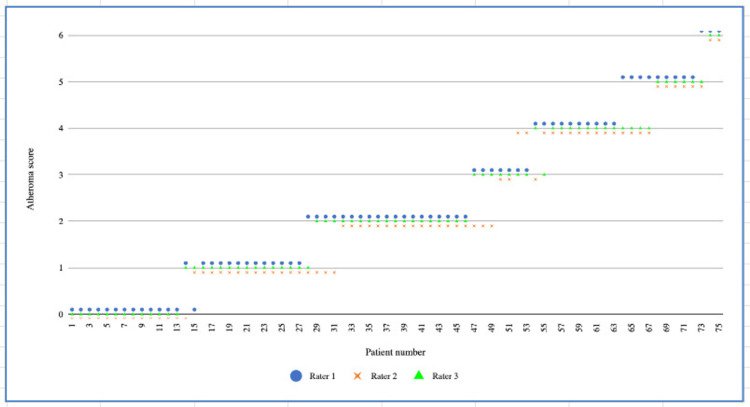
Graph demonstrating the atheroma score agreement between raters for each patient. Patients are ordered with increasing rater scores along the x-axis. The y-axis represents the total score (maximum score = 6).

There was excellent agreement between the three radiologists when using our tool for atheroma scoring, with ICC value = 0.972, (95% CI 0.959-0.981, p < 0.0001). Figure 3 depicts an ICC plot graph demonstrating the relative similarity of scores between raters. 

**Figure 4:**
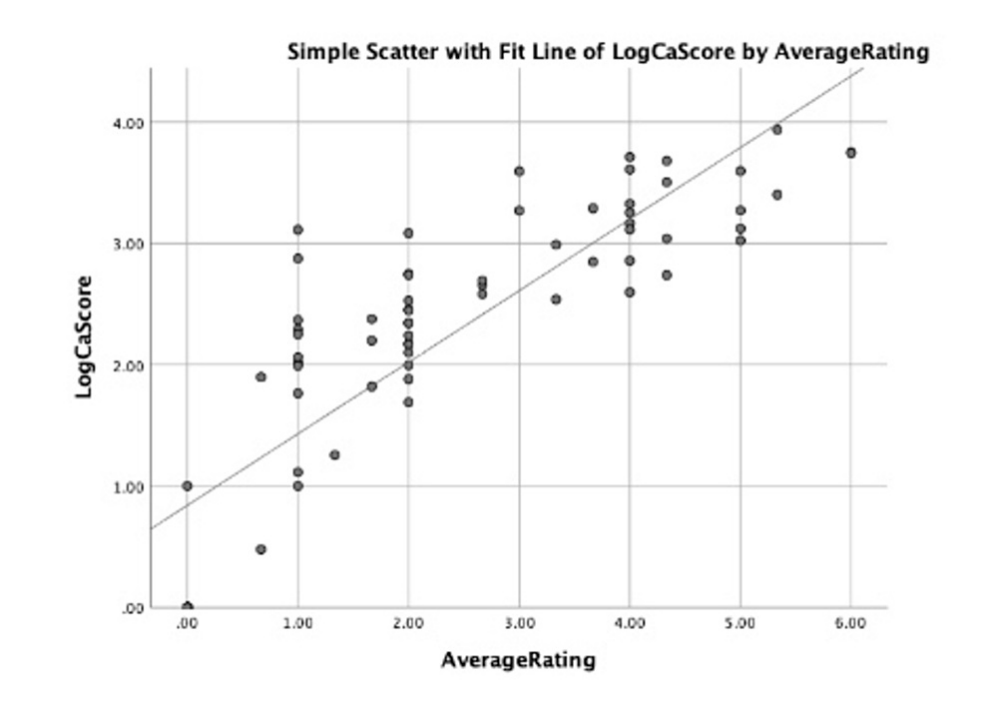
Graph depicting logarithmic data with a line of best fit showing the positive correlation between average rater atheroma score (x-axis) and corresponding calcium score (Log-scale on the y-axis).

There was also a strong correlation between the calculated CT calcium scores and the average rater score for each patient, Spearman rho = 0.894 (p < 0.0001). This is shown in the graph below (Figure 4).

The average time (per patient for the three radiologists) taken to calculate an atheroma score using the tool compared to the corresponding calcium scoring was recorded. The atheroma tool took an average of four minutes, whereas the calcium score took an average of 11 minutes (excluding the time taken to load the patient images from the PACS system to the vascular workstation, which otherwise would have further increased this time). This proves that the devised scoring tool is a time-saving method.

## Discussion

This study has shown that the devised novel AAC tool is a simple, reproducible, and time-saving semiquantitative method for grading the severity of AAC when compared to standard contemporaneous calcium scoring. This tool also correlates well with scores generated through formal calcium scoring. 

CT calcium scoring can be time-consuming in clinical practice and requires specific software and a degree of previous experience to interpret findings. Furthermore, as explained, it is not always possible to select segments of the aorta where there are confluent areas of calcification, resulting in the overestimation of disease for a determined length of the vessel. The AAC tool described in this study addresses these limitations. 

Only male patients were included as they had already undergone CTA for a different purpose: assessment of vascular disease and anatomy prior to prostate artery embolization. Hence it was suitable to perform a retrospective analysis using this patient cohort who already had appropriate imaging. 

There is no reason to suspect that gender would influence results or the way in which scores are calculated. The role which gender plays in disease severity is outside the scope of the study. The selected patient cohort had a wide range of disease severity, hence it was feasible to assess the reliability, reproducibility, and consistency of the scoring tool against different degrees of atherosclerosis. 

Although only midline vascular disease was measured and it can be argued that patients who have the eccentric disease would have an underestimated score, the statistical analysis proved a positive correlation between the AAC tool and corresponding formal calcium scoring (in which entire circumferential plaque is measured) regardless of disease severity. 

For reproducibility purposes, the segment of the abdominal aorta between the IMA and iliac bifurcation was scored. However, the scoring tool can be applied to any vessel that has minimal tortuosity.

## Conclusions

It is likely that there will be increasing research investigating the link between atheroma severity and risk stratification in numerous disease processes, for example, technical outcomes and re-intervention rates post endovascular aortic repair. The tool presented in this paper can be used as a simple method to evaluate and score AAC for these purposes, as well as for radiologists who simply want to comment on the degree of disease encountered during routine and emergency reporting sessions.

In conclusion, a simple, reproducible, and relatively fast system using CT to grade abdominal calcific atherosclerotic disease severity has been successfully demonstrated, with many anticipated potential uses in both research and clinical practice.
